# A comparative study benchmarking colon polyp with computer‐aided detection (CADe) software

**DOI:** 10.1002/deo2.70061

**Published:** 2025-01-18

**Authors:** Nikolaos Papachrysos, Pia Helén Smedsrud, Kim V. Ånonsen, Tor Jan D. Berstad, Håvard Espeland, Andreas Petlund, Per J. Hedenström, Pål Halvorsen, Jonas Varkey, Hugo L. Hammer, Michael A. Riegler, Thomas de Lange

**Affiliations:** ^1^ Department of Medicine, Geriatrics and Emergencies Division of Gastroenterology Sahlgrenska University Hospital/Östra Gothenburg Sweden; ^2^ Department of Molecular and Clinical Medicine, Institute of Medicine University of Gothenburg Gothenburg Sweden; ^3^ Augere Medical AS Oslo Norway; ^4^ Department of Informatics University of Oslo Oslo Norway; ^5^ SimulaMet Oslo Norway; ^6^ Department of Gastroenterology Oslo University Hospital Oslo Norway; ^7^ Department of Specialized Medicine Division of Gastroenterology Sahlgrenska University Hospital Gothenburg Sweden; ^8^ Oslo Metropolitan University Oslo Norway; ^9^ Department of Medicine & Emergencies Sahlgrenska University Hospital/Mölndal, Västra Götaland County Gothenburg Sweden

**Keywords:** artificial intelligence, CADe, colon polyps, colorectal cancer, polyp detection

## Abstract

**Background and aims:**

Computer‐aided detection software (CADe) has shown promising results in real‐time polyp detection, but a limited head‐to‐head comparison of the available CADe systems has been performed. Moreover, such systems have not been compared to endoscopists using standardized videos. This study aims to compare the performance of three CADe systems in detecting polyps, employing a novel standardized methodology.

**Methods:**

Videos from 300 colonoscopies conducted at Oslo University Hospital were analyzed. Short video clips (20–45 s) presenting normal mucosa or polyps were randomly selected. These videos were then streamed through each CADe system from Medtronic, Olympus, and Augere Medical. Each system featured diverse configurations, resulting in a total of six software settings. Sensitivity and false positivity (FP) were assessed by comparing the CADe systems to both the mean of the systems and pairwise between them. Furthermore, the systems’ performance was compared to the performance of five endoscopists.

**Results:**

CADe systems’ sensitivity ranged between 84.9% and 98.7%, with statistically significant differences observed between the systems, both in comparison to the mean and to each other. FP rates ranged between 1.2% and 5.6%, also differing statistically significantly between the systems. The CADe systems achieving the highest sensitivity also exhibited the highest FP. Statistically significant differences in the alert delay were observed between different CADe systems and endoscopists.

**Conclusions:**

This study highlights significant differences between commercially available CADe software regarding sensitivity and FP, but a superior performance compared to endoscopists. The software with the highest sensitivity also exhibited the highest FP, highlighting the need for further refinement.

## INTRODUCTION

Colorectal cancer (CRC) remains one of the most prevalent cancers globally.^1^, ^2^ Numerous studies confirm that high‐quality colonoscopy significantly reduces CRC incidence and mortality by detecting and removing premalignant lesions.^3^, ^4^ The adenoma detection rate (ADR) is a well‐studied and widely accepted key performance indicator for colonoscopy.^5^, ^6^, ^7^ A recent meta‐analysis of tandem colonoscopies demonstrated a miss rate between 9% and 27%.^8^ More than half of post‐colonoscopy CRC cases occur due to missed neoplastic lesions during index colonoscopy.^9^, ^10^ Various factors, such as procedural skills, perceptual factors, and knowledge deficits, may contribute to missed lesions.^11^, ^12^, ^13^ The European Society of Gastrointestinal Endoscopy recommends a target ADR of at least 25%.^14^ In comparison, the American Gastroenterology Association recommends an ADR of at least 30%, with an aspirational target of ≥35%.^15^ However, one study has suggested the benefits of increasing ADR above 50%.^16^

Currently, artificial intelligence is employed to develop software for real‐time colonic polyp detection, referred to as computer‐aided detection (CADe) systems. Numerous in‐silico studies on CADe systems have shown promising results.^3^, ^4^ These systems have also been studied in clinical trials during real‐time colonoscopy.^17^, ^18^, ^19^, ^20^ Several meta‐analyses enhance these findings, confirming that CADe‐assisted colonoscopy can increase endoscopists’ ADR up to 50%.^21^, ^22^ However, false positive alerts may distract the endoscopist, potentially leading to alarm fatigue or overdiagnosis of polyps.^21^, ^23^ According to a study by Hassan et al., false positivity (FP) was primarily attributed to artifacts from the intestinal wall, resulting in a 1% increase in the total withdrawal time.^24^

To date, there is no standard method for benchmarking and comparing different CADe software, and only a few studies have investigated differences in both sensitivity and FP, as well as how software settings influence various parameters.^25^ We performed the current study to create a publicly available dataset possible to use for later comparisons of software, and as such, increase the robustness of the comparisons. In our study, we benchmarked and compared CADe software from three manufacturers (Medtronic, Olympus, and Augere Medical) in a standardized way. Additionally, we compared the systems’ versus endoscopists’ performance in assessing the same videos.

## METHODS

### Study setting

**FIGURE 1:**
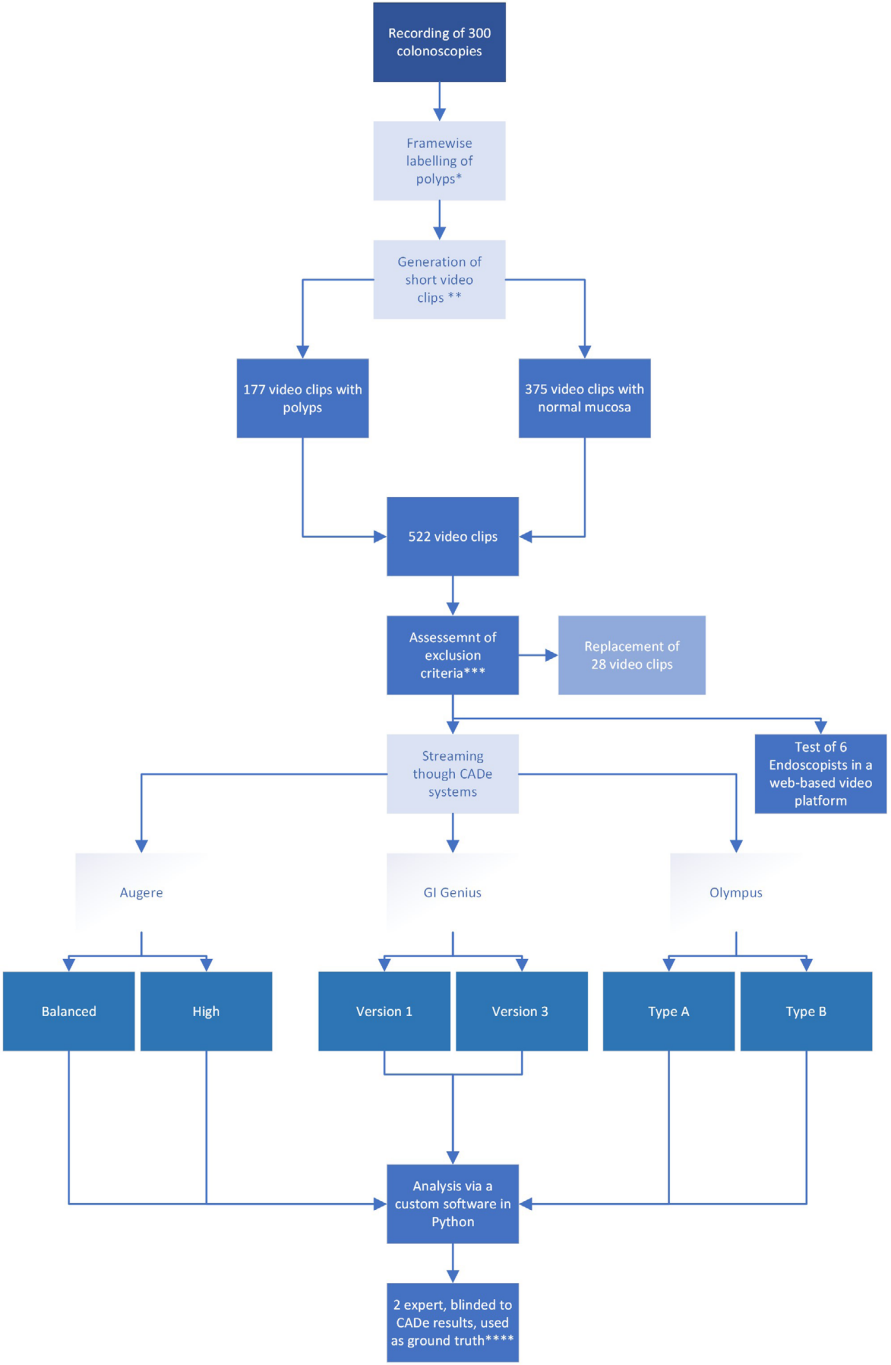
Study design flowchart (*labeling was done by trained healthcare students, **25–40 s, ***performed by an experienced gastroenterologist, **** in cases of disagreement between the two experts, the computer‐aided detection (CADe) polyp was reviewed by three experts in a physical meeting).

The flowchart (Figure 1) summarizes the study design. The detailed procedure for registering metadata, recording colonoscopy videos, and anonymizing the videos is provided in the Supporting Information.

#### Selection and validation of video clips

Consecutive patients referred to Oslo University Hospital due to bowel symptoms or for surveillance colonoscopy during the period November 15, 2019–January 23, 2020, were invited to participate in the study.

**FIGURE 2:**
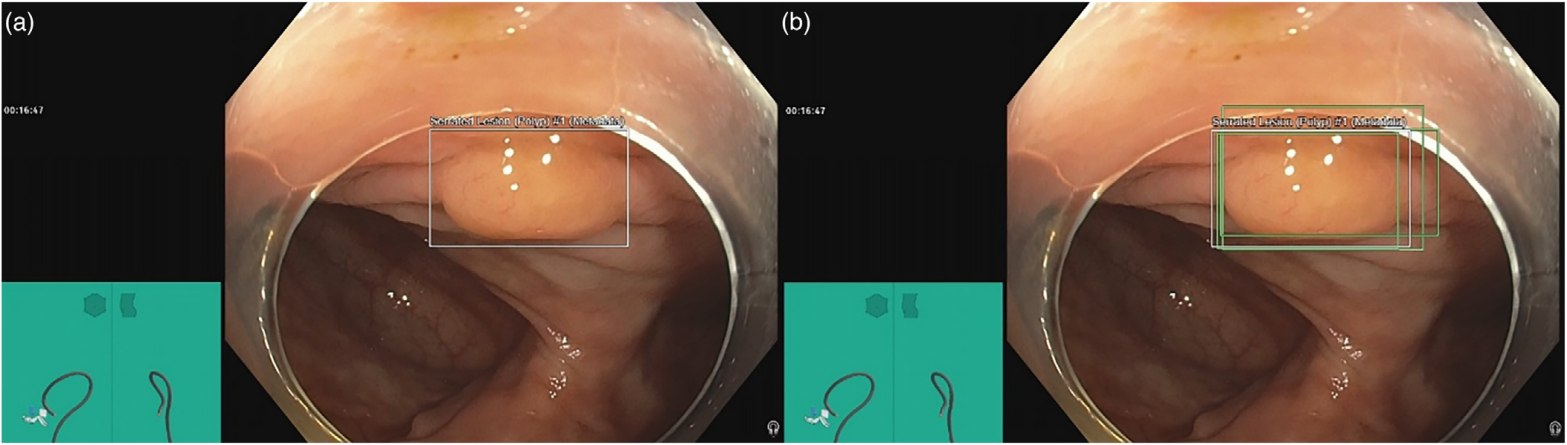
(A) SSL polyp detected by the performing endoscopist marked with a white bounding box, (B) Same polyp detected also by different types of computer‐aided detection software (green bounding boxes)

After labeling the original colonoscopy videos as described in the flowchart (Figure 1) and in the Supporting Information, short video clips of 25–40 s were automatically generated (Figure 2A). The video clips were generated in a way that the clip showed only normal mucosa or polyps in a 2:1 ratio. In clips with polyps, one detected polyp appeared at a random point in the clip, at least 3 s from the beginning and 5 s from the end of the clip. The average duration of polyps in the positive clips was 11.52 s (35.66%).

We excluded video clips with visible blood (except a minimal amount of blood after biopsy), accessories (e.g., biopsy forceps, and polyp snare), polyp resection area, small bowel mucosa, blurred or blocked mucosa for more than 50% of the clip, Boston Bowel Preparation Scale <2, or two detected polyps with a temporal distance of less than 3 s. Excluded video clips were replaced with new clips to achieve our initial target. Finally, an experienced gastroenterologist reviewed the video clips, to assess whether all exclusion criteria were considered, and if the polyps were correctly labeled with bounding boxes.

#### Test of three CADe polyp detection software

The selected video clips were stored on a Solid‐State Drive, played using a HyperDeck Studio 4K Pro video recorder (Blackmagic design), and streamed through the three CADe systems: GI Genius software versions 1.1 and 3.0 (Medtronic), EndoAid settings A and B (Olympus), and PolypAid settings balanced and high (Augere Medical). The CADe system's output, with bounding boxes overlay alerting for potential polyps, was recorded on a second Hyper‐Deck recorder (Figure 2B).

To analyze the videos, custom software was developed in Python (Python Software Foundation), using the Python Imaging Library, Open Computer Vision Library, and Scikit–an image processing library. Polyps detected by the CADe software were labeled ‘CADe polyps’ and verified by three expert endoscopists (>2000 colonoscopies) as either true polyps or false positive findings, using an annotation tool. The experts were blinded regarding which CADe software had detected the polyp. Each clip was reviewed by at least two experts. In cases of disagreement between the two experts, the CADe polyp was reviewed by the three experts in a physical meeting to reach a consensus on the findings and establish a valid ground truth. This process determined whether the CADe polyp was a polyp detected during the colonoscopy, a missed polyp, or a false positive finding. If the experts confirmed a previously undetected polyp, it was recorded as a ‘missed polyp’ in the database.

For the CADe systems, we considered it a true positive detection, accepting a delay of ≤20 frames (0.4 s) from when the polyp appeared or disappeared to compensate for the processing delay. For the endoscopists, we considered it a true positive detection if they pressed the spacebar within 1 second after the polyp disappeared from the video clip.

The custom comparison software was also utilized to analyze the performance in locating polyps in terms of the difference between the ground truth bounding box and the respective CADe software bounding box.

## TESTING ENDOSCOPISTS’ POLYP DETECTION

To compare the performance of CADe software and endoscopists, six endoscopists were invited to assess the same video clips analyzed by the CADe software in a web‐based video player. The endoscopist observers had performed from <300 to >1000 colonoscopies to represent a variety of potential users of a polyp CADe system. The ADR for the endoscopists ranged from 24,3% to 45.7%. One of the endoscopists was excluded due to technical problems and failed instructions. The endoscopists’ ability to detect polyps and their reaction time was measured. The process involved launching each video clip, with the option to stop it by pressing the spacebar if a polyp was observed. Once stopped, the video could not be rewound or watched further.

### Statistical analysis

The diagnostic yield of the CADe software was automatically analyzed in terms of sensitivity per polyp detected, specificity, and FP (1 – specificity) per frame. We considered FP to be more relevant in assessing false alerts with the potential to distract endoscopists and reduce their trust in the system. Specificity and FP were based on frame‐wise statistics and calculated as the proportion of normal frames alerted as polyp. To avoid overconfident statistical tests when comparing the FP of two CADe systems, we accounted for the strong dependency in the prediction outcome for subsequent frames by estimating the autocorrelation in identification between consecutive frames.

The sensitivity of the software, according to polypwise statistics, was based on whether the observer (endoscopist or CADe system) detected a polyp or not. Sensitivity was calculated in the same manner for both endoscopists and the CADe systems. We performed two types of comparisons: one to the mean sensitivity of the software or of the endoscopists, and a pairwise comparison of all systems and endoscopists. When comparing the CADe system or endoscopist, there were some differences since the endoscopists in some cases stopped the video clip early if a polyp was detected. Therefore, to compare if there were statistically significant differences in sensitivity, a combination of paired and unpaired comparisons was performed using a linear mixed‐effect model. To assess whether the sensitivity of the observers in detecting polyps depended on the characteristics of the polyp, sensitivity was analyzed for four polyp groups; all polyps, polyps initially detected by the endoscopist, polyps missed by the endoscopist, and histologically verified SSLs.

## RESULTS

### Baseline results

We recorded 300 colonoscopy videos, and each colonoscopy video generated at least one short video clip. All patients scheduled for colonoscopy were included in the study upon providing consent. Demographic details and baseline results are shown in Table 1.

**TABLE 1:** Baseline characteristics

Patients aged ≥45 years	236 (79%)
BBPS 2–3 in all segments	287 (96%)
BBPS 0–1 in any segment	13 (4%)
Adenoma detection rate	23%
Polyp detection rate	43%
Adenoma/colonoscopy rate	0.65
Colorectal cancers	4 (1%)

A total of 20 endoscopists performed the colonoscopies, with the number of colonoscopies per endoscopist ranging from 1 to 49. The endoscopists exhibited diverse levels of experience, ranging from fellows in training to those who had performed more than five thousand colonoscopies.

The verification process of the short video clips led to the replacement of 28 clips. The final study dataset comprised 522 video clips of whom 177 contained polyps and 375 without polyps.

### Sensitivity

**FIGURE 3:**
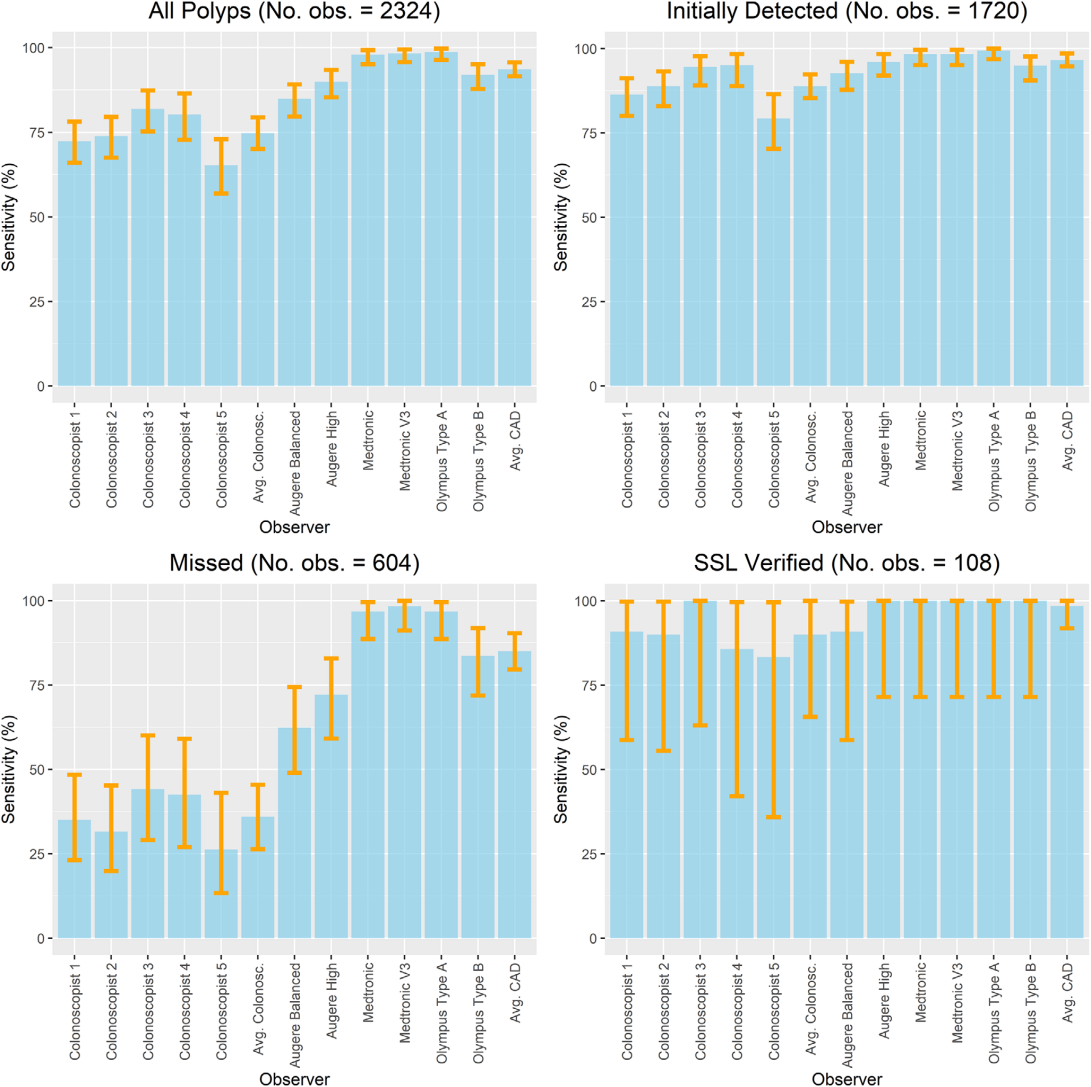
Sensitivity per finding for the endoscopists and computer‐aided detection systems. The panels, from upper left to lower right, display the polyp groups: all polyps, initially detected polyps, missed polyps, and histologically verified SSLs. The bars ‘Avg. Colonosc.’ indicate the average sensitivity for the five endoscopists. The orange intervals represent 95% confidence intervals for sensitivity estimates, and the *p*‐values for pair‐wise comparisons are provided in Tables SA1–A4.

There was a statistically significant difference in the sensitivity of various CADe systems ranging from 84.9% to 98.7%. Similarly, we detected a statistically significant difference in the endoscopists' sensitivity for polyp detection ranging from 65.3% to 81.9% (Table 2). As depicted in Table 2 and Figure 3, CADe systems' sensitivity for polyp detection was superior to the endoscopist's sensitivity. A detailed presentation of pairwise comparisons of the CADe systems and endoscopists' sensitivity is shown in Supporting Information.

**TABLE 2:** Sensitivity for polyp detection

Observer	Sensitivity	95% CI	*p*‐value
Augere balanced	84.9%	79.7%–89.2%	<0.0001
Augere high	89.9%	85.4%–93.4%	<0.0001
Medtronic 1.1	97.9%	95.2%–99.3%	0.0005
Medtronic 3.0	98.3%	95.8%–99.5%	<0.0001
Olympus type A	98.7%	96.4%–99.7%	<0.0001
Olympus type B	92.0%	87.8%–95.1%	0.0774
AVG. CADe	**93.6%**	**91.5%–95.7%**	**<0.0001**
Endoscopist 1	72.4%	66.0%–78,2%	0.0806
Endoscopist 2	73.9%	67.5%–79.6%	0.1560
Endoscopist 3	81.9%	75.3%–87.3%	0.0002
Endoscopist 4	80.3%	72.8%–86.5%	0.0021
Endoscopist 5	65.3%	56.9%–73.0%	<0.0001
AVG. Endoscopists	**74.7%**	**70.1%–79.4%**	

*Note*: The *p*‐value compares the average sensitivity of the CADe systems to the individual system and the average sensitivity of the endoscopist to the individual endoscopist. The *p*‐value in the row ‘Average CADe’ compares the average sensitivity of the CADe systems and the average sensitivity of the endoscopists.

### False positivity

**FIGURE 4:**
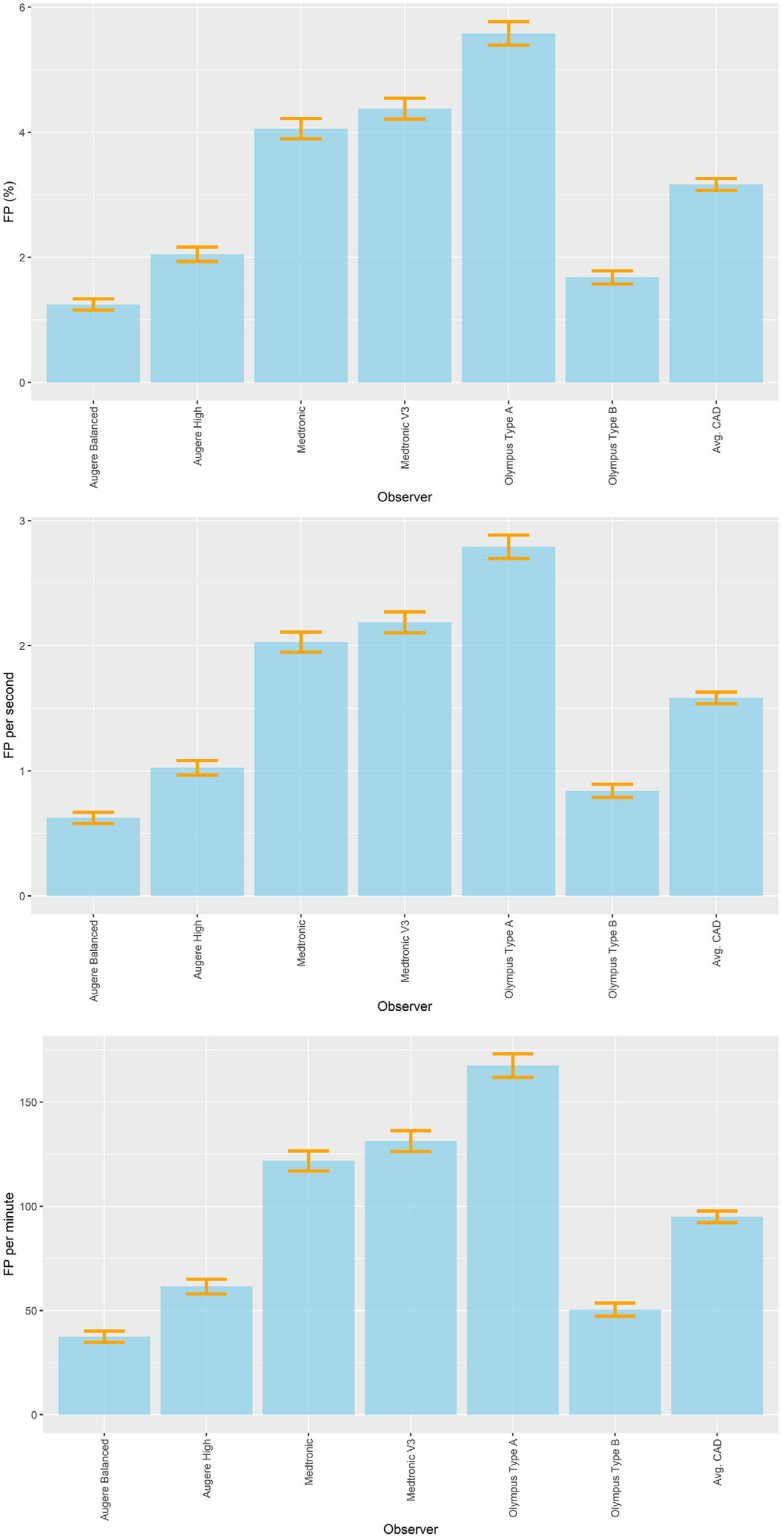
Framewise false positivity (FP) for the different computer‐aided detection (CADe) systems %, per second, and per minute. The orange intervals represent 95% confidence intervals. The p‐values for pair‐wise comparison can be found in Tables SA5–A8.

There were statistically significant differences in FP between the CADe systems ranging from 1.2% to 5.6% with an average FP of 3.2% (95% confidence interval [CI]: 3.1%–3.3%), (Table 3 and Figure 4). Augere Balanced setting had the lowest FP of 1.2% (95% CI: 1.1%–1.3%) while Olympus Type A had the highest FP of 5.6% (95% CI: 5.4%–5.8%).

**TABLE 3:** False positivity for polyp detection

Observer	FP	95% CI
Augere balanced	1.2%	1.1–1.3%
Augere high	2.1%	1.9–2.2%
Medtronic 1.1	4.1%	3.9–4.2%
Medtronic 3.0	4.4%	4.2–4.5%
Olympus type A	5.6%	5.4–5.8%
Olympus type B	1.7%	1.6–1.8%
AVG. CAD	**3.2%**	**3.1%–3.3%**

### Alert‐delay

**FIGURE 5:**
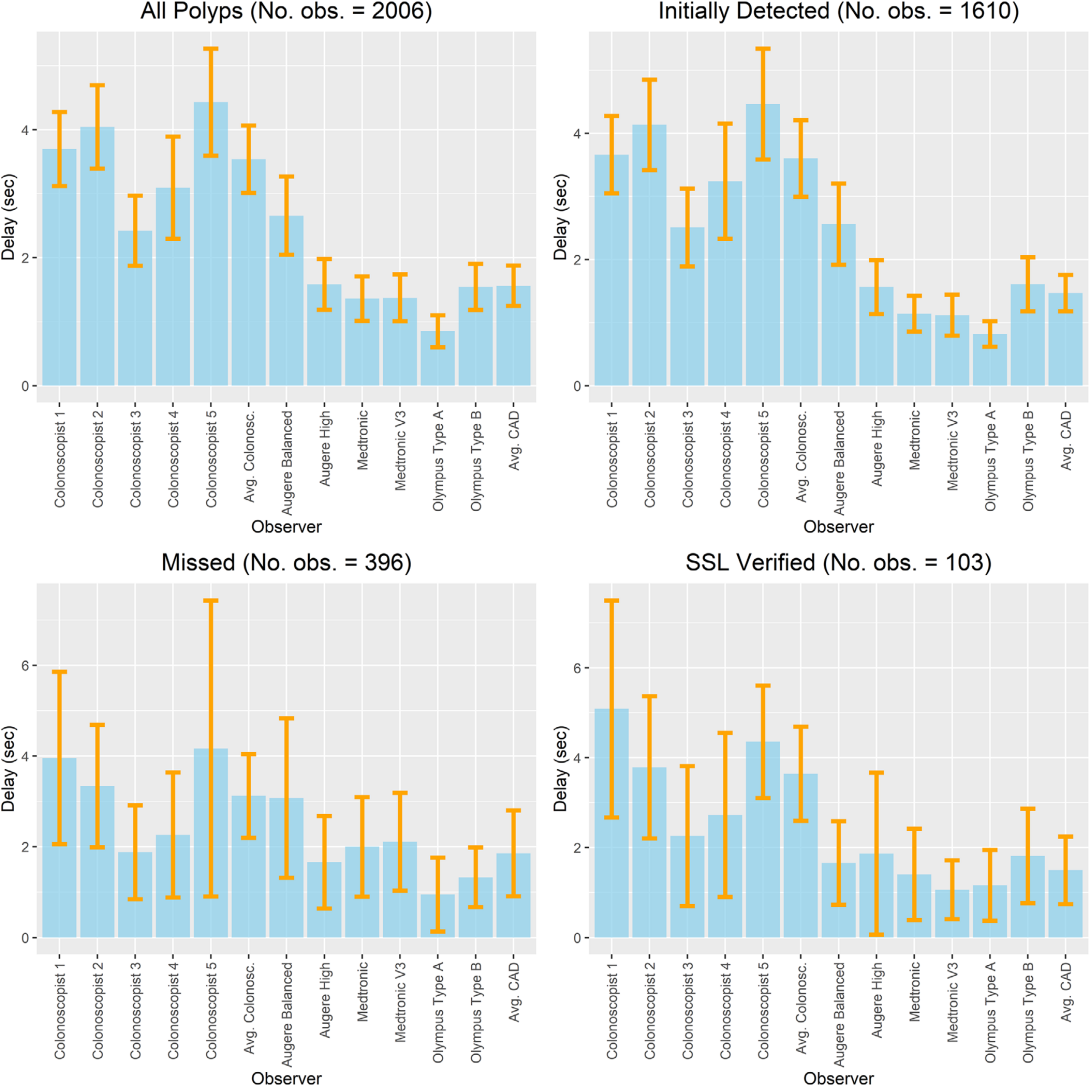
Average delay in seconds between the first frame with a polyp and the time the endoscopist alerted the detection of the polyp or the computer‐aided detection (CADe) system's first alerting bounding box appeared. The p‐values for pair‐wise comparison can be found in Tables SA5–A8.

The average delay between the first polyp‐positive frame and its detection by either the CADe system (first frame with bounding box) or the endoscopists (triggered by pressing the spacebar) exhibited statistically significant variations. The delay IQR ranged from 0.04 to 5.64 s (5%–95% quantile) with a median of 0.48 s in CADe systems and from 0.65 to 11.86 s (5%–95% quantile) with a median of 2.20 s among endoscopists. Furthermore, the alert delay varied based on the nature of the polyp (Figure 5). Detailed analyses on the polyp detection delay for both endoscopists and CADe systems are shown in the Supporting Information.

## DISCUSSION

We have established a standardized methodology to compare and benchmark CADe software for polyp detection. To the best of our knowledge, this is the first study to perform such a head–to–head comparison, including a comparison to endoscopists.

Our study demonstrates statistically significant differences between the systems. The three CADe software with the highest sensitivity were Olympus Type A and Medtronic versions 1 and 3, but they also exhibited the highest FP, increasing the risk of alert fatigue. However, the settings of two of the systems may be adjusted at the endoscopists’ discretion. As expected, the CADe systems performed better than the endoscopists, both with respect to polyp detection and reaction delay. Additionally, there were statistically significant differences between the endoscopists.

Overall, all the CADe systems achieved a very high sensitivity of 90%–100%. While sensitivity is crucial, there is a trade‐off between high sensitivity and a high FP rate. This balance can have consequences for the endoscopists' workflow, and the clinical relevance of the findings. Numerous false alerts may disturb endoscopists, leading to alarm fatigue and potentially increasing examination time due to unnecessary checking of alert sites or removal of insignificant mucosal abnormalities, as highlighted in recent systematic reviews.^26^, ^27^ Additionally, it can distract endoscopists and reduce their enthusiasm for CADe. In a recent randomized controlled trial, the withdrawal time was not different when using a highly sensitive CADe system compared to a standard colonoscopy.^28^ However, it's important to emphasize that the study included only highly skilled screening endoscopists. Conversely, Hassan et al. demonstrated a 1% increase in colonoscopy withdrawal time, primarily due to artifacts from the bowel wall.^24^ In a recent study it was shown that the effectiveness of CADe appears to decrease when the FP rate exceeds 5 FPs per minute, as this diminishes its clinical value.^29^

Several actions may be taken to enhance the sensitivity and FP of CADe software. One approach is to increase and diversify the amount of video footage in the image database used to train the software. Further fine‐tuning of the algorithms should be conducted, with a specific focus on addressing false alerts. This can be achieved by e.g. adjusting the thresholds for probability predicted by the artificial intelligence model^30^ or the number of frames with suspected polyps before the system triggers an alert.^31^ These adjustments aim to optimize the software's performance and find a balance between sensitivity and specificity, ultimately improving its clinical utility and user‐friendliness. A recent study showed that post‐update improvements in CADe systems led to a reduction in false positives without impairing polyp detection, potentially alleviating the burden on endoscopists.^32^

Recent studies have emphasized the impact of CADe systems in clinical practice. A pivotal study in this field by Repici et al.^33^ demonstrated that ADR was significantly higher in the CADe group compared to the control group (54.8% vs. 40.4%). CADe detected a notably higher proportion of diminutive (<5 mm) and small adenomas (6–9 mm). However, no difference was found in the detection rate of larger polyps, a finding also supported by a more recent prospective study from Japan.^34^ According to a recent meta‐analysis,^35^ CADe is the most promising technique to increase ADR above 40% when compared with alternative techniques like chromoendoscopy. Furthermore, a recent randomized controlled trial^36^ demonstrated that combining CADe with a mucosal exposure device can improve ADR up to 16.8%, surpassing the impact of using each technique separately.

The primary concern with the use of CADe systems is that the effect of CADe on improving the detection of advanced adenomas may be smaller in comparison to the detection of diminutive and small polyps. Two studies, one prospective with nearly eight years of follow‐up on diminutive polyps,^37^ and a systematic review on the natural history of small and diminutive polyps,^38^ demonstrated the benign character of such polyps. Although, up to 6% of those polyps had progressed to advanced adenomas. Neither study found cases of CRC, but the follow–up periods were too short to detect potential differences.

These findings, in addition to our results, highlight the benefit of using polyp CADe software to assist endoscopists in detecting polyps to achieve an expert level. This not only contributes to preventing CRC to the same extent but also ensures equitable healthcare for all patients. While most studies in this field involve experienced endoscopists, the potential ADR increase with CADe may be more substantial for younger endoscopists or trainees. Furthermore, CADe can be valuable in assisting experienced and skilled endoscopists during moments of inattention, such as fatigue at the end of the day. However, the actual impact on endoscopists’ workload, stress, and clinical decision‐making is not fully assessed in the literature.

The three CADe systems tested in this study have some technical differences but are all designed for use with Olympus endoscopy video processors. GI‐Genius is compatible with both EVIS EXERA III and EVIS X1 processors but cannot process 4K video signals. EndoAid is exclusively compatible with EVIS X1. Finally, PolypAid is compatible with both EVIS X1 and EXERA III and can also process 4K video signals.

The video footage analyzed in this study represents a diverse selection of samples found in daily practice, including examinations performed by highly skilled endoscopists and trainees, and increases the generalizability of the results. However, it is important that all recordings are from a single center, and a further extension of the database with recordings from several centers could potentially improve the validity of the tests. The CADe systems were tested on Olympus endoscopy video processors. The results may not be directly applicable to systems from other manufacturers or processors. Another limitation is that the validity has a relatively short half‐life since CADe software could be continuously updated and improved. Despite this, no significant changes were observed in the results for GI Genius version 1.1 and 3.0. Lastly, our study evaluates technical aspects, without exploring the clinical impact of CADe systems, such as patient outcomes or long‐term benefits.

In conclusion, our study demonstrates the efficiency of polyp CADe software, which outperforms endoscopists in terms of polyp detection and detection delay. However, significant differences exist among different CADe software, highlighting the need for improvement, particularly in achieving a better balance between sensitivity and FP. This emphasizes the potential for further advancements in CADe technology to enhance its overall performance and contribute to more accurate and reliable polyp detection during colonoscopies.

POCACO: Polyp Videos for CADe Comparison,

DOI: https://doi.org/10.5281/zenodo.14444296

## CONFLICT OF INTEREST STATEMENT

Authors Pia Helén Smedsrud, Tor Jan D. Berstad, and Thomas de Lange are employed and shareholders in Augere Medical. Håvard Espeland, Andreas Petlund, Pål Halvorsen, and Michael A. Riegler are shareholders in Augere Medical.

## ETHICS STATEMENT

Approval of the research protocol by an Institutional Reviewer Board; The study received approval from the Data Inspectorate of Southeast Norway and was exempted for ethical approval since it did not interfere with patient treatment (18/17158, 09.11.2018).

## PATIENT CONSENT STATEMENT

If not applicable; All included patients provided signed informed consent, and the study adhered to the principles outlined in the Declaration of Helsinki, and according to good clinical practice. Additionally, all video clips obtained during the study were anonymized.

## CLINICAL TRIAL REGISTRATION

N/A.

## Supporting information




**TABLE SA1** Pairwise comparison of sensitivity for all polyps. C1–C5 refers to Endoscopists 1–5, AB and AH to Augere Medical setting Balanced and High, MV1 and MV3 to Medtronic version 1.1 and Medtronic Version 3.0 and OTA and OTB to Olympus Setting A and B. The values represent the difference in sensitivity (percentage) and the colors denote the level of statistical significance and are Bonferroni‐corrected for the number of tests (*n* = 55); that is, dark blue denotes *p*‐value <0.001/55, blue denotes *p*‐value <0.01/55 and light blue denotes *p*‐value <0.05/55.
**TABLE SA2** Pairwise comparison of sensitivity for initially detected polyps. C1–C5 refers to Endoscopists 1–5, AB and AH to Augere Balanced and High, MV1 and MV3 to Medtronic version 1.1 and Medtronic Version 3.0 and OTA and OTB to Olympus Type A and B. The values represent the difference in sensitivity (percentage) and the colors denote the level of statistical significance and are Bonferroni‐corrected for the number of tests (*n* = 55); that is, dark blue denotes *p*‐value <0.001/55, blue denotes *p*‐value <0.01/55 and light blue denotes *p*‐value <0.05/55.
**TABLE SA3** Pairwise comparison of sensitivity for missed polyps. C1–C5 refers to Endoscopists 1–5, AB and AH to Augere Balanced and High, MV1 and MV3 to Medtronic version 1.1 and Medtronic Version 3.0 and OTA and OTB to Olympus Type A and B. The values represent the difference in sensitivity (percentage) and the colors denote the level of statistical significance and are Bonferroni‐corrected for the number of tests (*n* = 55); that is, dark blue denotes *p*‐value <0.001/55, blue denotes *p*‐value <0.01/55 and light blue denotes *p*‐value <0.05/55.
**TABLE SA4** Pairwise comparison of sensitivity for histology‐verified SSLs. C1–C5 refers to Endoscopists 1–5, AB and AH to Augere Balanced and High, MV1 and MV3 to Medtronic version 1.1 and Medtronic Version 3.0 and OTA and OTB to Olympus Type A and B. The values represent the difference in sensitivity (percentage) and the colors denote the level of statistical significance and are Bonferroni‐corrected for the number of tests (*n* = 55); that is, dark blue denotes *p*‐value <0.001/55, blue denotes *p*‐value <0.01/55 and light blue denotes *p*‐value <0.05/55.
**TABLE SA5** Pairwise comparison of average delay for all polyps. C1–C5 refers to Endoscopists 1–5, AB and AH to Augere Balanced and High, MV1 and MV3 to Medtronic version 1.1 and Medtronic Version 3.0 and OTA and OTB to Olympus Type A and B. The values represent the difference in delay (in seconds) and the colors denote the level of statistical significance and are Bonferroni‐corrected for the number of tests (*n* = 55); that is, dark blue denotes *p*‐value <0.001/55, blue denotes *p*‐value <0.01/55, and light blue denotes *p*‐value <0.05/55.
**TABLE SA6** Pairwise comparison of average delay for initially detected polyps. C1–C5 refers to Endoscopists 1–5, AB and AH to Augere Balanced and High, MV1 and MV3 to Medtronic version 1.1 and Medtronic Version 3.0 and OTA and OTB to Olympus Type A and B. The values represent the difference in delay (in seconds) and the colors denote the level of statistical significance and are Bonferroni‐corrected for the number of tests (*n* = 55); that is, dark blue denotes *p*‐value <0.001/55, blue denotes *p*‐value <0.01/55, and light blue denotes *p*‐value <0.05/55.
**TABLE SA7** Pairwise comparison of average delay for missed polyps. C1–C5 refers to Endoscopists 1–5, AB and AH to Augere Balanced and High, MV1 and MV3 to Medtronic version 1.1 and Medtronic Version 3.0 and OTA and OTB to Olympus Type A and B. The values represent the difference in delay (in seconds) and the colors denote the level of statistical significance and are Bonferroni‐corrected for the number of tests (*n* = 55); that is, dark blue denotes *p*‐value <0.001/55, blue denotes *p*‐value <0.01/55, and light blue denotes *p*‐value <0.05/55.
**TABLE SA8** Pairwise comparison of average delay for histology‐verified SSLs. C1–C5 refers to Endoscopists 1–5, AB and AH to Augere Balanced and High, MV1 and MV3 to Medtronic version 1.1 and Medtronic Version 3.0 and OTA and OTB to Olympus Type A and B. The values represent the difference in delay (in seconds) and the colors denote the level of statistical significance and are Bonferroni‐corrected for the number of tests (*n* = 55); that is, dark blue denotes *p*‐value <0.001/55, blue denotes *p*‐value <0.01/55, and light blue denotes *p*‐value <0.05/55.
