# Supplementary material for: A comparative study benchmarking colon polyp with computer‐aided detection (CADe) software
Source: DEN Open. 2025 Jan 18;5(1):e70061. doi: 10.1002/deo2.70061 (PMC11742239; doi:10.1002/deo2.70061)
Supplement: Supplementary file 1 — TABLE SA1 Pairwise comparison of sensitivity for all polyps. C1–C5 refers to Endoscopists 1–5, AB and AH to Augere Medical setting Balanced and High, MV1 and MV3 to Medtronic version 1.1 and Medtronic Version 3.0 and OTA and OTB to Olympus Setting A and B. The values represent the difference in sensitivity (percentage) and the colors denote the level of statistical significance and are Bonferroni‐corrected for the number of tests (n = 55); that is, dark blue denotes p‐value <0.001/55, blue denotes p‐value <0.01/55 and light blue denotes p‐value <0.05/55. TABLE SA2 Pairwise comparison of sensitivity for initially detected polyps. C1–C5 refers to Endoscopists 1–5, AB and AH to Augere Balanced and High, MV1 and MV3 to Medtronic version 1.1 and Medtronic Version 3.0 and OTA and OTB to Olympus Type A and B. The values represent the difference in sensitivity (percentage) and the colors denote the level of statistical significance and are Bonferroni‐corrected for the number of tests (n = 55); that is, dark blue denotes p‐value <0.001/55, blue denotes p‐value <0.01/55 and light blue denotes p‐value <0.05/55. TABLE SA3 Pairwise comparison of sensitivity for missed polyps. C1–C5 refers to Endoscopists 1–5, AB and AH to Augere Balanced and High, MV1 and MV3 to Medtronic version 1.1 and Medtronic Version 3.0 and OTA and OTB to Olympus Type A and B. The values represent the difference in sensitivity (percentage) and the colors denote the level of statistical significance and are Bonferroni‐corrected for the number of tests (n = 55); that is, dark blue denotes p‐value <0.001/55, blue denotes p‐value <0.01/55 and light blue denotes p‐value <0.05/55. TABLE SA4 Pairwise comparison of sensitivity for histology‐verified SSLs. C1–C5 refers to Endoscopists 1–5, AB and AH to Augere Balanced and High, MV1 and MV3 to Medtronic version 1.1 and Medtronic Version 3.0 and OTA and OTB to Olympus Type A and B. The values represent the difference in sensitivity (percentage) and the colors denote [file DEO2-5-e70061-s001.docx]

# Supplementary materials

## Methods Study setting

The initial steps of the study are described in detail below.

### Video recording of colonoscopies

The examinations were performed with Evis Exera III™ systems and CF/PCF 190 HQi™ colonoscopes (Olympus, Tokyo, Japan). The entire examination including intubation and withdrawal was video recorded in a video format with a Pix–E5, 4K Video recorder, (Sound Devices, LLC USA).

The colonoscopies were performed by the endoscopist of various experience assigned to perform it according to the endoscopy service ordinary scheduling. Specific variables were recorded in a dedicated study database by a study nurse during each colonoscopy, with instructions to record all polyps whether they were resected or not. When the videos and all variables were recorded the data were anonymized and exported to an external server in batches of 6 for further processing.

### Labelling of videos

The recorded colonoscopy videos and corresponding metadata were imported to a proprietary software developed by Augere Medical for framewise labelling of the polyps. All polyps detected by the performing endoscopists were labelled by bounding boxes (small rectangles). We have developed an electronic labelling tool providing the possibility to play the endoscopy videos frame by frame, both forwards and backwards and at the same time annotate the polyp with a bounding box. The bounding box is placed by the annotators on the first frame at which polyp is at least partially visible, for technical and user experience reasons there is a limitation that the bounding box size must be at least 50 pixels on each side.

Polyps were first labelled by trained healthcare students according to the time codes recorded for detected polyps during the colonoscopy. These polyps detected by the performing endoscopist, are named initially detected polyps. At the first frame a detected polyp appeared, the healthcare student started to label the polyp with a bounding box, and they continued to add bounding boxes for a maximum of 2000 frames (40 seconds at 50 frames per second (fps)) if the polyp did not disappear before. If the polyp disappeared and reappeared before the 2000 frames were labelled, the additional frames were labelled until 2000 frames were reached or until the polyp disappeared for good.

## Additional Statistical Analyses

Tables A1 to A4 show pairwise comparison in sensitivity between the endoscopists and the CADe systems for the polyp categories: all polyps (A1), initially detected polyps (A2), missed polyps (A3) and histology verified SSLs (A4). A positive value means that a method indicated at the top of the column had a higher detection rate (sensitivity) than the method indicated by the row. For example, from the first row we see that AB has a 12.6% higher detection rate than C1.

Tables A5 to A8 show pairwise comparison in average delay between the endoscopists and the CADe systems for the polyp categories: all polyps (A5), initially detected polyps (A6), missed polyps (A7) and histology-verified SSLs (A8). A positive value means that a method indicated at the top of the column had a higher delay than the method indicated by the row.

**Table A1:** Pairwise comparison of sensitivity for all polyps. C1 to C5 refer to Endoscopist 1 to 5, AB and AH to Augere Medical setting Balanced and High, MV1 and MV3 to Medtronic version 1.1 and Medtronic Version 3.0 and OTA and OTB to Olympus Setting A and B. The values represent the difference in sensitivity (percentage) and the colors denote the level of statistical significance and are Bonferroni-corrected for the number of tests (*n* = 55), i.e. dark blue denotes p–value < 0.001/55, blue denotes p–value < 0.01/55 and light blue denotes p–value < 0.05/55.

|  | **C2** | **C3** | **C4** | **C5** | **AB** | **AH** | **MV1** | **MV3** | **OTA** | **OTB** |
| --- | --- | --- | --- | --- | --- | --- | --- | --- | --- | --- |
| **C1** | 0.8 | 8.2 | 8.7 | –5.8 | 12.6 | 17.4 | 25.5 | 26 | 26.3 | 19.4 |
| **C2** | – | 8.6 | 6.9 | –7.4 | 10.8 | 15.9 | 24 | 24.5 | 24.9 | 18.1 |
| **C3** | – | – | –1 | –16.8 | 2.7 | 7.5 | 16.1 | 16.4 | 16.8 | 10.1 |
| **C4** | – | – | – | –15.1 | 3.4 | 8.5 | 17.6 | 18 | 18.3 | 11.6 |
| **C5** | – | – | – | – | 19 | 24.3 | 32.6 | 33 | 33.4 | 26.8 |
| **AB** | – | – | – | – | – | 5.0 | 13 | 13.4 | 13.9 | 7.1 |
| **AH** | – | – | – | – | – | – | 8 | 8.4 | 8.8 | 2.1 |
| **M** | – | – | – | – | – | – | – | 0.4 | 0.8 | –5.9 |
| **MV3** | – | – | – | – | – | – | – | – | 0.4 | –6.3 |
| **OTA** | – | – | – | – | – | – | – | – | – | –6.7 |

**Table A2:** Pairwise comparison of sensitivity for initially detected polyps. C1 to C5 refer to Endoscopist 1 to 5, AB and AH to Augere Balanced and High, MV1 and MV3 to Medtronic version 1.1 and Medtronic Version 3.0 and OTA and OTB to Olympus Type A and B. The values represent the difference in sensitivity (percentage) and the colors denote the level of statistical significance and are Bonferroni-corrected for the number of tests (*n* = 55), i.e. dark blue denotes p–value < 0.001/55, blue denotes p–value < 0.01/55 and light blue denotes p–value < 0.05/55.

|  | **C2** | **C3** | **C4** | **C5** | **AB** | **AH** | **MV1** | **MV3** | **OTA** | **OTB** |
| --- | --- | --- | --- | --- | --- | --- | --- | --- | --- | --- |
| **C1** | 2.2 | 7.5 | 8.8 | –6.1 | 6.9 | 9.8 | 12 | 12 | 13.1 | 8.4 . |
| **C2** | – | 6 | 6.2 | –9.3 | 4.1 | 7.3 | 9.5 | 9.5 | 10.6 | 6.1 |
| **C3** | – | – | 0.3 | –15.4 | –1.6 | 1.5 | 3.8 | 3.8 | 4.9 | 0.3 |
| **C4** | – | – | – | –15.2 | –2.5 | 0.8 | 3.1 | 3.1 | 4.2 | –0.2 |
| **C5** | – | – | – | – | 13.4 | 16.7 | 19.1 | 19.1 | 20.2 | 15.8 |
| **AB** | – | – | – | – | – | 3.4 | 5.6 . | 5.6 . | 6.8 | 2.3 |
| **AH** | – | – | – | – | – | – | 2.3 | 2.3 | 3.4 | –1.1 |
| **M** | – | – | – | – | – | – | – | 0 | 1.1 | –3.4 |
| **MV3** | – | – | – | – | – | – | – | – | 1.1 | –3.4 |
| **OTA** | – | – | – | – | – | – | – | – | – | –4.5 |

**Table A3:** Pairwise comparison of sensitivity for missed polyps. C1 to C5 refer to Endoscopist 1 to 5, AB and AH to Augere Balanced and High, MV1 and MV3 to Medtronic version 1.1 and Medtronic Version 3.0 and OTA and OTB to Olympus Type A and B. The values represent the difference in sensitivity (percentage) and the colors denote the level of statistical significance and are Bonferroni-corrected for the number of tests (*n* = 55), i.e. dark blue denotes p–value < 0.001/55, blue denotes p–value < 0.01/55 and light blue denotes p–value < 0.05/55.

|  | **C2** | **C3** | **C4** | **C5** | **AB** | **AH** | **M** | **MV3** | **OTA** | **OTB** |
| --- | --- | --- | --- | --- | --- | --- | --- | --- | --- | --- |
| **C1** | –3.5 | 9.2 | 8.7 | –7.8 | 27.1 | 37 | 61.7 | 63.4 | 61.7 | 48.5 |
| **C2** | – | 14 | 10.4 | –3.5 | 29.9 | 39.9 | 65.1 | 66.8 | 65.1 | 51.8 |
| **C3** | – | – | –0.4 | –18.2 | 15.5 | 25.6 | 52.6 | 54.1 | 52.4 | 39.4 |
| **C4** | – | – | – | –17.7 | 17.3 | 27.5 | 54.2 | 55.8 | 54 | 41 |
| **C5** | – | – | – | – | 34.8 | 45.4 | 70.2 | 72 | 70.2 | 57.4 |
| **AB** | – | – | – | – | – | 9.8 | 34.4 | 36.1 | 34.4 | 21.3 |
| **AH** | – | – | – | – | – | – | 24.6 | 26.2 | 24.6 | 11.5 |
| **M** | – | – | – | – | – | – | – | 1.6 | 0 | –13.1 |
| **MV3** | – | – | – | – | – | – | – | – | –1.6 | –14.8 |
| **OTA** | – | – | – | – | – | – | – | – | – | –13.1 |

**Table A4:** Pairwise comparison of sensitivity for histology verified SSLs. C1 to C5 refer to Endoscopist 1 to 5, AB and AH to Augere Balanced and High, MV1 and MV3 to Medtronic version 1.1 and Medtronic Version 3.0 and OTA and OTB to Olympus Type A and B. The values represent the difference in sensitivity (percentage) and the colors denote the level of statistical significance and are Bonferroni-corrected for the number of tests (*n* = 55), i.e. dark blue denotes p–value < 0.001/55, blue denotes p–value < 0.01/55 and light blue denotes p–value < 0.05/55.

|  | **C2** | **C3** | **C4** | **C5** | **AB** | **AH** | **MV1** | **MV3** | **OTA** | **OTB** |
| --- | --- | --- | --- | --- | --- | --- | --- | --- | --- | --- |
| **C1** | 0 | 0 | –5.2 | –7.6 | 0 | 9.1 | 9.1 | 9.1 | 9.1 | 9.1 |
| **C2** | – | 0 | –4.3 | –6.7 | 0 | 10 | 10 | 10 | 10 | 10 |
| **C3** | – | – | –14.3 | –16.7 | 0 | 0 | 0 | 0 | 0 | 0 |
| **C4** | – | – | – | –2.4 | 5.2 | 14.3 | 14.3 | 14.3 | 14.3 | 14.3 |
| **C5** | – | – | – | – | 7.6 | 16.7 | 16.7 | 16.7 | 16.7 | 16.7 |
| **AB** | – | – | – | – | – | 9.1 | 9.1 | 9.1 | 9.1 | 9.1 |
| **AH** | – | – | – | – | – | – | 0 | 0 | 0 | 0 |
| **M** | – | – | – | – | – | – | – | 0 | 0 | 0 |
| **MV3** | – | – | – | – | – | – | – | – | 0 | 0 |
| **OTA** | – | – | – | – | – | – | – | – | – | 0 |

**Table A5**: Pairwise comparison of average delay for all polyps. C1 to C5 refer to Endoscopist 1 to 5, AB and AH to Augere Balanced and High, MV1 and MV3 to Medtronic version 1.1 and Medtronic Version 3.0 and OTA and OTB to Olympus Type A and B. The values represent the difference in delay (in seconds) and the colors denote the level of statistical significance and are Bonferroni-corrected for the number of tests (*n* = 55), i.e. dark blue denotes p–value < 0.001/55, blue denotes p–value < 0.01/55 and light blue denotes p–value < 0.05/55.

|  | **C2** | **C3** | **C4** | **C5** | **AB** | **AH** | **MV1** | **MV3** | **OTA** | **OTB** |
| --- | --- | --- | --- | --- | --- | --- | --- | --- | --- | --- |
| **C1** | 0.26 | –1.54 | –1.09 | 0.56 | –1.44 | –2.27 | –2.43 | –2.5 | –2.87 | –2.2 |
| **C2** | – | –1.41 | –1.03 | 0.14 | –1.54 | –2.56 | –2.75 | –2.81 | –3.2 | –2.48 |
| **C3** | – | – | 0.47 | 1.77 | –0.05 | –0.97 | –1.12 | –1.14 | –1.59 | –0.95 |
| **C4** | – | – | – | 1.68 | –0.63 | –1.53 | –1.74 | –1.76 | –2.25 | –1.58 |
| **C5** | – | – | – | – | –1.6 | –2.69 | –3.07 | –3.12 | –3.57 | –2.81 |
| **AB** | – | – | – | – | – | –1.11 | –1.39 | –1.41 | –1.82 | –1.17 |
| **AH** | – | – | – | – | – | – | –0.39 | –0.36 | –0.78 | –0.04 |
| **M** | – | – | – | – | – | – | – | 0.01 | –0.5 | 0.31 |
| **MV3** | – | – | – | – | – | – | – | – | –0.51 | 0.32 |
| **OTA** | – | – | – | – | – | – | – | – | – | 0.75 |

**Table A6**: Pairwise comparison of average delay for initially detected polyps. C1 to C5 refer to Endoscopist 1 to 5, AB and AH to Augere Balanced and High, MV1 and MV3 to Medtronic version 1.1 and Medtronic Version 3.0 and OTA and OTB to Olympus Type A and B. The values represent the difference in delay (in seconds) and the colors denote the level of statistical significance and are Bonferroni-corrected for the number of tests (*n* = 55), i.e. dark blue denotes p–value < 0.001/55, blue denotes p–value < 0.01/55 and light blue denotes p–value < 0.05/55.

|  | **C2** | **C3** | **C4** | **C5** | **AB** | **AH** | **MV1** | **MV3** | **OTA** | **OTB** |
| --- | --- | --- | --- | --- | --- | --- | --- | --- | --- | --- |
| **C1** | 0.29 | –1.42 | –0.95 | 0.59 | –1.37 | –2.23 | –2.54 | –2.6 | –2.85 | –2.11 |
| **C2** | – | –1.39 | –0.98 | 0.13 | –1.71 | –2.67 | –3.01 | –3.05 | –3.31 | –2.53 |
| **C3** | – | – | 0.56 | 1.77 | –0.06 | –1.02 | –1.39 | –1.43 | –1.69 | –0.97 . |
| **C4** | – | – | – | 1.6 | –0.65 | –1.69 | –2.09 | –2.14 | –2.42 | –1.65 |
| **C5** | – | – | – | – | –1.69 | –2.75 | –3.28 | –3.36 | –3.63 | –2.8 |
| **AB** | – | – | – | – | – | –1.05 | –1.44 | –1.49 | –1.75 | –1.02 |
| **AH** | – | – | – | – | – | – | –0.43 | –0.46 | –0.76 | 0.05 |
| **M** | – | – | – | – | – | – | – | –0.03 | –0.33 | 0.47 |
| **MV3** | – | – | – | – | – | – | – | – | –0.3 | 0.5 |
| **OTA** | – | – | – | – | – | – | – | – | – | 0.8 |

**Table A7**: Pairwise comparison of average delay for missed polyps. C1 to C5 refer to Endoscopist 1 to 5, AB and AH to Augere Balanced and High, MV1 and MV3 to Medtronic version 1.1 and Medtronic Version 3.0 and OTA and OTB to Olympus Type A and B. The values represent the difference in delay (in seconds) and the colors denote the level of statistical significance and are Bonferroni-corrected for the number of tests (*n* = 55), i.e. dark blue denotes p–value < 0.001/55, blue denotes p–value < 0.01/55 and light blue denotes p–value < 0.05/55.

|  | **C2** | **C3** | **C4** | **C5** | **AB** | **AH** | **MV1** | **MV3** | **OTA** | **OTB** |
| --- | --- | --- | --- | --- | --- | --- | --- | --- | --- | --- |
| **C1** | –0.02 | –2.08 | –2.04 | 0.42 | –1.67 | –2.56 | –2.53 | –2.52 | –3.01 | –2.65 |
| **C2** | – | –1.56 | –1.31 | –0.27 | –0.24 | –1.58 | –2.1 | –2.06 | –2.64 | –1.86 |
| **C3** | – | – | 0.38 | 1.59 | –0.61 | –0.57 | 0.12 | 0.23 | –1.39 | –0.55 |
| **C4** | – | – | – | 2.21 | –0.89 | –0.75 | –0.2 | –0.12 | –1.68 | –0.93 |
| **C5** | – | – | – | – | –1.04 | –2.24 | –2.38 | –2.33 | –3.22 | –2.65 |
| **AB** | – | – | – | – | – | –1.38 | –1.43 | –1.31 | –2.15 | –1.79 |
| **AH** | – | – | – | – | – | – | –0.48 | –0.32 | –0.81 | –0.42 |
| **M** | – | – | – | – | – | – | – | 0.12 | –0.97 | 0.01 |
| **MV3** | – | – | – | – | – | – | – | – | –1.08 | –0.12 |
| **OTA** | – | – | – | – | – | – | – | – | – | 0.72 |

**Table A8**: Pairwise comparison of average delay for histology verified SSLs. C1 to C5 refer to Endoscopist 1 to 5, AB and AH to Augere Balanced and High, MV1 and MV3 to Medtronic version 1.1 and Medtronic Version 3.0 and OTA and OTB to Olympus Type A and B. The values represent the difference in delay (in seconds) and the colors denote the level of statistical significance and are Bonferroni-corrected for the number of tests (*n* = 55), i.e. dark blue denotes p–value < 0.001/55, blue denotes p–value < 0.01/55 and light blue denotes p–value < 0.05/55.

|  | **C2** | **C3** | **C4** | **C5** | **AB** | **AH** | **MV1** | **MV3** | **OTA** | **OTB** |
| --- | --- | --- | --- | --- | --- | --- | --- | --- | --- | --- |
| **C1** | –1.54 | –2.89 | –2.64 | –1.14 | –3.43 | –3.67 | –3.77 | –4.06 | –3.91 | –3.31 |
| **C2** | – | –1.53 | –0.96 | 0.39 | –2.1 | –2.46 | –2.42 | –2.75 | –2.61 | –1.98 |
| **C3** | – | – | 0.48 | 2.57 | –0.6 | –0.39 | –0.85 | –1.19 | –1.09 | –0.44 |
| **C4** | – | – | – | 1.67 | –1.07 | –1.18 | –1.31 | –1.63 | –1.53 | –0.9 |
| **C5** | – | – | – | – | –2.54 | –2.63 | –2.84 | –3.22 | –2.99 | –2.34 |
| **AB** | – | – | – | – | – | –0.46 | –0.35 | –0.66 | –0.46 | 0.09 |
| **AH** | – | – | – | – | – | – | –0.46 | –0.81 | –0.71 | –0.05 |
| **M** | – | – | – | – | – | – | – | –0.34 | –0.24 | 0.41 |
| **MV3** | – | – | – | – | – | – | – | – | 0.1 | 0.75 |
| **OTA** | – | – | – | – | – | – | – | – | – | 0.65 |
